# 靶向CLL-1嵌合抗原受体T细胞的构建及其功能验证

**DOI:** 10.3760/cma.j.issn.0253-2727.2022.02.003

**Published:** 2022-02

**Authors:** 笑 柴, 鑫 金, 明峰 赵

**Affiliations:** 天津市第一中心医院，天津 300192 Tianjin First Central Hospital, Tianjin 300192, China

**Keywords:** 白血病，髓样，急性, C型凝集素样分子1, 嵌合抗原受体, 细胞治疗, Leukemia, myeloid, acute, C-type lectin-like molecule 1, Chimeric antigen receptor, Cell therapy

## Abstract

**目的:**

探索开发一种靶向CLL-1的嵌合抗原受体T细胞（CAR-T细胞）并验证其功能。

**方法:**

通过流式细胞术检测急性髓系白血病（AML）细胞系和AML原代细胞CLL-1靶点的表达水平。构建CLL-1 CAR载体并制备出相应慢病毒，感染激活后T细胞生产出CAR-T细胞，并通过体外和体内实验验证CLL-1 CAR-T细胞的功能。

**结果:**

AML细胞系和原代AML细胞中均表达CLL-1。制备的CLL-1 CAR-T细胞转导率为77.82％，在AML细胞系以及AML原代细胞中，CLL-1 CAR-T细胞能明显特异性杀伤表达CLL-1的靶细胞系和原代肿瘤细胞。相对于未转导的T细胞，CLL-1 CAR-T细胞在杀伤靶细胞和原代肿瘤细胞时分泌IL-6、IFN-γ等细胞因子水平更高（*P*值均<0.001）。在AML人源性异种移植小鼠模型中，相对于未转导的T细胞，CLL-1 CAR-T细胞表现出有效的抗白血病活性并延长小鼠存活时间［未达到对22（95％ *CI* 19～24）d，*P*＝0.002］。

**结论:**

靶向CLL-1的CAR-T细胞成功开发并具有较好的肿瘤杀伤作用。

## Abstract (en)

**Objective:**

To explore the development of a CAR-T cells targeting CLL-1 and verify its function.

**Methods:**

The expression levels of CLL-1 targets in cell lines and primary cells were detected by flow cytometry. A CLL-1 CAR vector was constructed, and the corresponding lentivirus was prepared. After infection and activation of T cells, CAR-T cells targeting CLL-1 were produced and their function was verified in vitro and in vivo.

**Results:**

CLL-1 was expressed in acute myeloid leukemia（AML）cell lines and primary AML cells. The transduction rate of the prepared CAR T cells was 77.82％. In AML cell lines and AML primary cells, CLL-1-targeting CAR-T cells significantly and specifically killed CLL-1-expressing cells. Compared to untransduced T cells, CAR-T cells killed target cells and secreted inflammatory cytokines, such as interleukin-6 and interferon-γ, at significantly higher levels（*P*<0.001）. In an in vivo human xenograft mouse model of AML, CLL-1 CAR-T cells also exhibited potent antileukemic activity and induced prolonged mouse survival compared with untransduced T cells［not reached *vs* 22 days（95％ *CI* 19–24 days）, *P*＝0.002］.

**Conclusion:**

CAR-T cells targeting CLL-1 have been successfully produced and have excellent functions.

急性髓系白血病（AML）是成人最常见的急性白血病类型，目前的主要治疗措施有化疗、基因靶向治疗和造血干细胞移植，复发难治是影响患者生存的重要因素[1]–[2]。嵌合抗原受体T细胞（CAR-T细胞）治疗是通过基因工程的方法将一种或多种特异性嵌合抗原受体（CAR）表达于T细胞使其可靶向作用于肿瘤细胞的过继免疫疗法[3]–[4]。CAR-T细胞免疫治疗是近年来肿瘤免疫治疗的里程碑，尤其在B细胞血液恶性肿瘤的治疗中取得了显著的疗效[5]。近年来越来越多的靶点用于AML的免疫治疗，如CD33、CD123、CLL-1、CD47、CD70和TIM3[6]–[16]。其中，CLL-1选择性在白血病干细胞（LSC）表面表达，而在正常造血干细胞中不表达，Morsink等[17]研究证实CLL-1是AML的理想靶标。本研究，我们开发了一种靶向CLL-1的CAR结构并进行了功能验证，现报道如下。

## 材料与方法

1. 细胞系和原代细胞：HEK-293T细胞培养于含10％胎牛血清（FBS）的Dulbecco改良Eagle培养基（DMEM），THP1、U937、MOLM13细胞培养于含10％ FBS的RPMI 1640培养基。培养基购自美国Gibco公司。所有细胞系均购于美国模式培养物集存库（ATCC），在37 °C、5％ CO_2_和95％湿度条件下培养。按照机构指南获得知情同意后，从本单位的AML患者骨髓和健康供者的外周血获取单个核细胞样本。

2. 质粒构建：靶向CLL-1的单链可变区片段（ScFv）序列来源于M26克隆。CLL-1 CAR结构由CLL-1 ScFv、CD8a铰链区和跨膜区、CD137共刺激信号、CD3ζ链胞内信号结构域组成，将CLL-1 CAR序列克隆到pCDH-MND-MCS-T2A-Puro骨架质粒中，从而获得慢病毒转移载体。慢病毒质粒的制备和滴度检测采用美国GeneCopoeia公司的试剂盒完成。

3. CAR-T细胞的制备：采用CD3免疫磁珠（德国Miltenyi公司）从外周血单核细胞中分离出CD3^+^ T细胞，使用CD3/CD28刺激磁珠和IL-2（美国Thermo Fisher公司）扩增T细胞。24 h后通过含有抗CLL-1 CAR基因的慢病毒质粒感染激活后的T细胞，然后在转导后第3天检测转导效率。

4. CLL-1 CAR-T细胞检测：将细胞样品用藻红蛋白（PE）标记的多克隆山羊抗小鼠IgG（H+L）抗体（美国Affinity公司产品）染色来检测CAR表达，使用Coulter Altra流式细胞仪（美国Beckman公司产品）配套的CytExpert软件进行分析。

5. 细胞的活性和增殖能力：取5×10^5^的细胞，离心后弃上清，加入100 µl PBS重悬细胞，加入5 µl碘化丙啶染色，室温孵育5 min后加入PBS清洗2次。通过流式细胞术分析细胞活性，计算细胞增殖率。

6. 细胞因子评估：效应细胞与靶细胞共孵育24 h后，收集24孔板中细胞离心后的上清液，用CBA试剂盒测定其细胞因子（包括IL-2、IL-6、IL-8、IL-10、TNF-α、IFN-γ）表达水平。使用NovoCyte流式细胞仪（Agilent）进行检测与分析。

7. 细胞毒性测定：采用GFP标记的肿瘤细胞与CLL-1 CAR-T细胞或未转导的T细胞按一定效靶比以相同体积和相同肿瘤细胞数量进行共孵育。24 h后，取一定体积的细胞，通过流式细胞术检测肿瘤细胞的数量，通过以下公式计算肿瘤细胞杀伤作用：（空白对照孔肿瘤细胞数-实验孔肿瘤细胞数）/空白对照孔肿瘤细胞数×100％。

8. 小鼠肿瘤模型：10只6～8周龄的雄性NSG小鼠购自斯贝福（北京）生物技术有限公司，随机分为两组，每组5只，通过尾静脉注射3×10^6^本实验室自行保存的荧光素酶表达的MOLM13细胞构建AML模型。3 d后，分别注入5×10^6^ CLL-1 CAR-T细胞和未转导的T细胞进行治疗。为检测小鼠肿瘤负荷，在指定的时间点，每只小鼠腹膜内注射3 mg D-荧光素（美国Sigma公司产品），并在10 min后使用IVIS Imager生物活体成像仪（德国PerkinElmer公司）对小鼠进行活体成像。

9. 统计学处理：使用GraphPad Prism进行统计学分析。服从正态分布的数据以均值±标准差表示，组间比较使用单因素方差分析或独立样本*t*检验。使用Kaplan-Meier法绘制生存曲线。*P*<0.05为差异有统计学意义。

## 结果

一、AML细胞系和原代AML细胞中CLL-1表达

为了验证CLL-1是针对AML的CAR-T细胞治疗的靶抗原，我们首先评估了AML细胞系和原代AML细胞中CLL-1的表达。结果显示，慢性髓性白血病细胞株K562细胞不表达CLL-1，将其用作阴性对照。几种不同强度的AML细胞系（THP1、U937、MOLM13）均表达CLL-1（图1）。我们进一步分析了10例初发AML患者（5例M_5_、3例M_2_、2例M_4_）的骨髓样本中原代AML细胞CLL-1的表达，大部分患者的AML细胞较高表达CLL-1（图1B）。

**图1:**
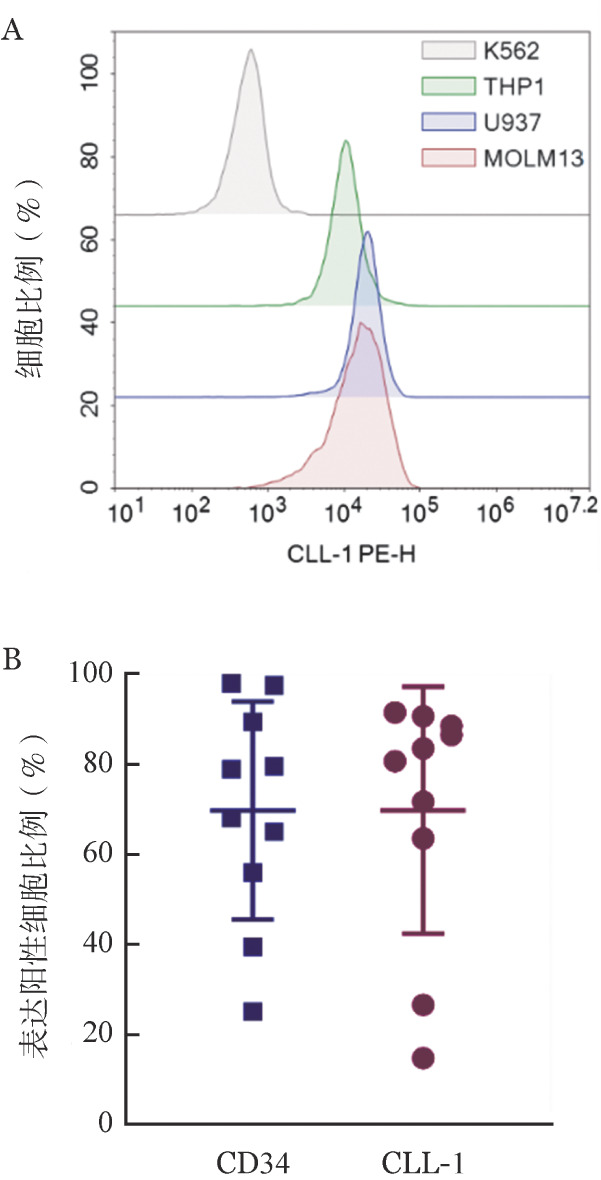
急性髓系白血病（AML）细胞系和原代AML细胞中CLL-1的表达水平 A：流式细胞术检测K562、THP1、U937、MOLM13细胞中CLL-1表达水平；B：10例初发AML患者原代AML细胞中CD34和CLL-1的阳性细胞比例

二、CLL-1 CAR-T细胞的制备

本研究设计的CLL-1 CAR结构由CLL-1 ScFv、CD8a铰链区和跨膜区、CD137共刺激信号、CD3ζ链胞内信号结构域组成（图2）。将制备的CLL-1 CAR-T细胞采用IgG（H+L）抗体标记，流式细胞仪检测显示CLL-1 CAR-T细胞转导率为77.82％，提示CLL-1 CAR-T细胞成功制备。

**图2:**
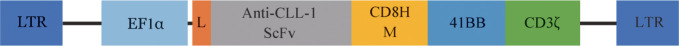
靶向CLL-1的嵌合抗原受体结构示意图

三、CLL-1 CAR-T功能验证

1. AML细胞系：将未转导的T细胞和CLL-1 CAR-T细胞分别与CLL-1阳性的MOLM13细胞和CLL-1阴性的K562细胞按不同效靶比共孵育。相比未转导的T细胞，CLL-1 CAR-T细胞可以明显杀伤MOLM13细胞，而对K562细胞没有杀伤作用（图3）。检测1∶1效靶比孵育后上清的细胞因子，IL-2、IL-4、IL-6、IL-10、IFN-γ、TNF-α仅在CLL-1 CAR-T细胞与MOLM13细胞共孵育组中有明显的上调（表1）。

**图3:**
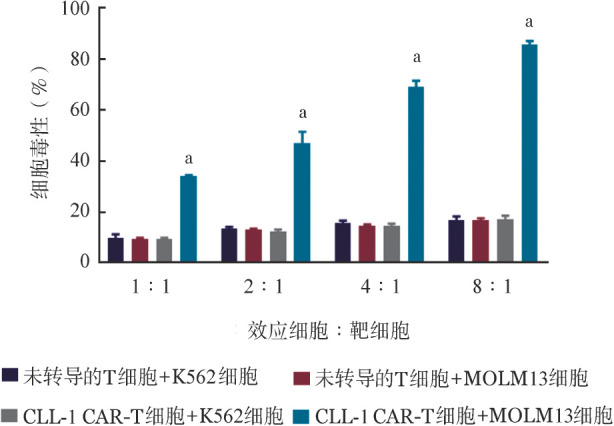
未转导的T细胞和CLL-1 CAR-T细胞分别与MOLM13和K562细胞按不同效靶比共孵育24 h后的细胞毒作用（实验重复3次，与其他各组比较^a^*P*<0.001） CLL-1 CAR-T细胞：靶向CLL-1的嵌合抗原受体T细胞

**表1:** 未转导的T细胞和CLL-1 CAR-T细胞分别与MOLM13和K562细胞按1∶1效靶比共孵育24 h后的细胞因子表达水平（ng/L，*x*±*s*）

组别	细胞因子水平					
	IL-2	IL-4	IL-6	IL-10	IFN-γ	TNF-α
未转导的T细胞+K562	2 099.34±90.59	21.55±1.81	14.95±0.39	23.19±0.60	1 336.82±27.93	28.28±0.81
未转导的T细胞+MOLM13	2 146.40±41.97	22.87±0.58	14.83±0.33	23.17±0.60	1 327.21±35.20	28.60±0.75
CLL-1 CAR-T+K562	2 145.84±57.70	21.10±1.18	14.49±0.05	22.73±0.13	1 301.75±6.80	27.64±0.66
CLL-1 CAR-T+MOLM13	14 401.94±416.31^a^	44.20±0.58 ^a^	41.46±2.21 ^a^	158.09±2.42 ^a^	4 770.12±154.09 ^a^	119.85±3.65 ^a^

注：CLL-1 CAR-T：靶向CLL-1的嵌合抗原受体T细胞。实验重复3次。与其他各组比较，^a^*P*<0.05

2. 原代AML细胞：将获得的CLL-1 CAR-T细胞与3例流式细胞术检测CLL-1表达超过90％的原代AML细胞按1∶1的效靶比共孵育。相比未转导的T细胞，CLL-1 CAR-T细胞可以明显杀伤原代AML细胞（图4A）。检测孵育后上清中IFN-γ的表达水平，相比未转导的T细胞，CLL-1 CAR-T细胞与原代AML细胞共孵育上清中的IFN-γ明显增加（图4B）。

**图4:**
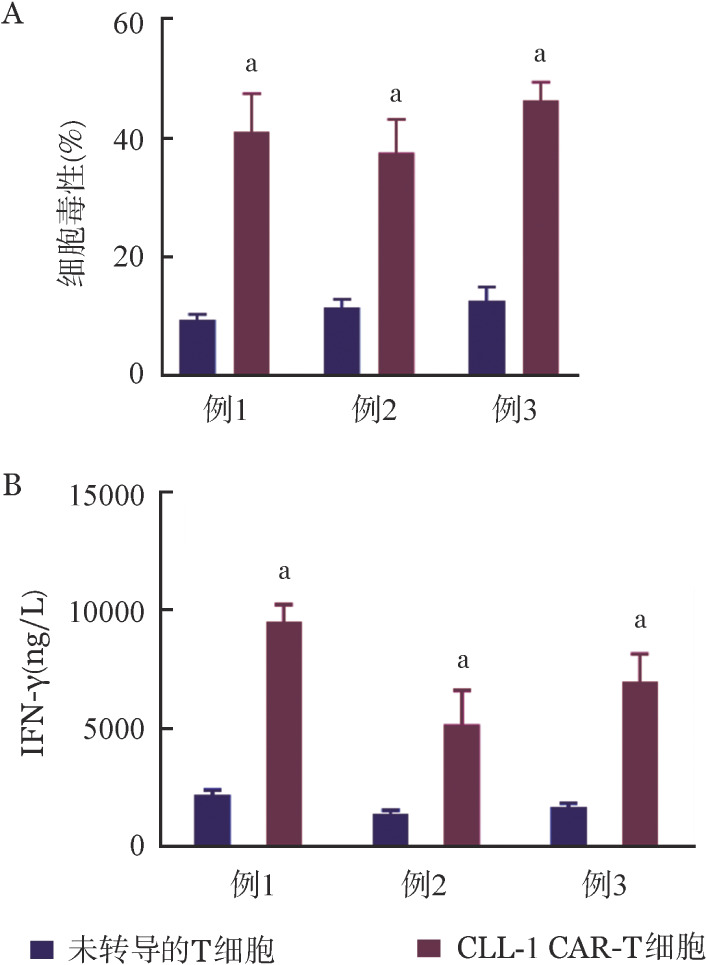
CLL-1 CAR-T细胞与3例CLL-1阳性的原代AML细胞按1∶1的效靶比共孵育24 h的杀伤作用（实验重复3次，^a^*P*<0.001） CLL-1 CAR-T：靶向CLL-1的嵌合抗原受体T细胞；AML：急性髓系白血病；A：细胞毒作用；B：IFN-γ表达水平

3. AML人源性异种移植小鼠模型：为了确认CLL-1 CAR-T细胞的体内抗白血病活性，我们使用了AML人源性异种移植小鼠模型。结果见图5，相比接受未转导的T细胞组，接受CLL-1 CAR-T细胞的小鼠显示出白血病负荷降低，中位存活时间显著延长［未达到对22（95％ *CI* 19～24）d，*P*＝0.002］。

**图5:**
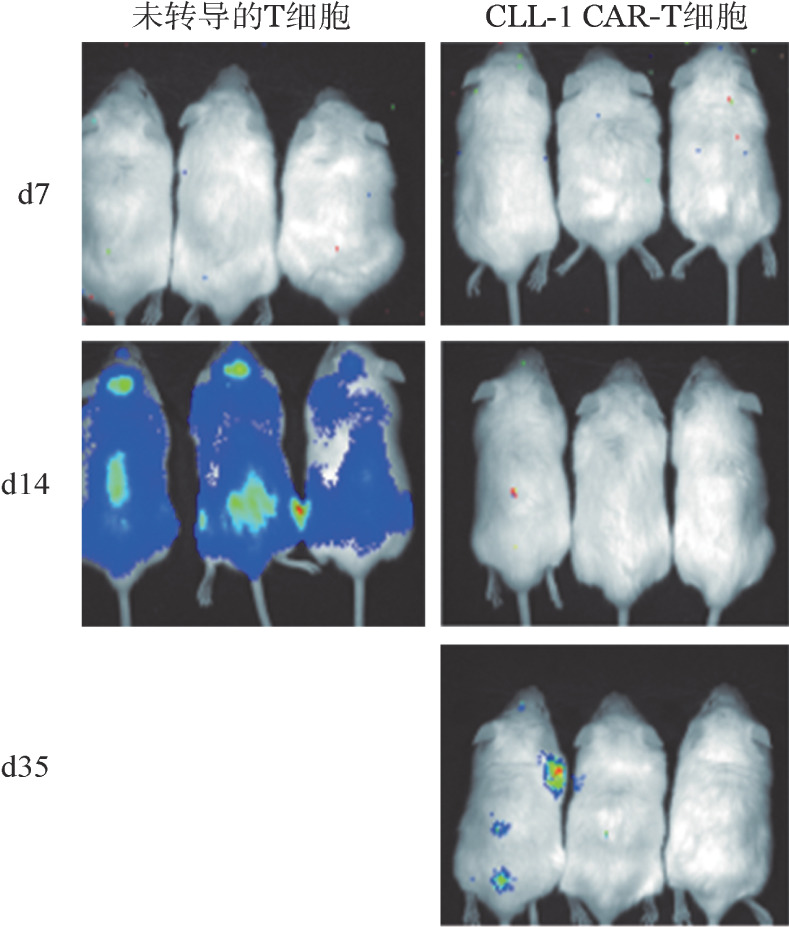
活体成像技术检测CLL-1 CAR-T细胞在AML人源性异种移植小鼠模型中的杀伤作用 CLL-1 CAR-T细胞：靶向CLL-1的嵌合抗原受体T细胞；AML：急性髓系白血病

## 讨论

CLL-1在AML细胞和LSC选择性表达，在非血液组织中不表达，为AML免疫治疗的理想靶标[15]–[17]。已有研究者开发和优化了用于AML的CLL-1 CAR-T细胞，并证实CLL-1 CAR-T细胞具有特异性抗白血病活性[10],[12],[18]。关于CLL-1 CAR-T的结构，Tashiro等[18]在比较CD28、4-1BB和OX40的一种或两种组合后，发现4-1BB具有最强的刺激T细胞产生特定细胞因子并维持持久细胞毒性的能力。我们在既往研究的基础上，构建了CLL-1 CAR载体，并制备出高转导效率的CAR-T细胞，其在AML细胞系和原代AML细胞表现出明显的细胞毒作用和细胞因子分泌；随后体内试验显示，在AML小鼠模型中，相对于未转导的T细胞，CLL-1 CAR-T细胞在体内表现出有效的抗白血病活性并延长小鼠存活时间。

安全性是CAR-T细胞研究中的关注重点，尽管CLL-1在正常HSC不表达，但成熟粒细胞高表达CLL-1，从而引发了对CLL-1 CAR-T细胞治疗造成长期粒细胞缺乏，从而易发感染的担忧[15]。在现有的探索性临床试验报道中，严重粒细胞缺乏是CLL-1 CAR-T细胞治疗的主要毒副作用之一[19]。为了解决这个问题，一些研究者采用了“开关型”的结构来控制CAR-T细胞毒性[20]–[21]。此外，CAR-T细胞对糖皮质激素具有高敏感性，糖皮质激素类药物对CAR-T细胞的杀伤作用可部分中和CAR-T治疗的相关毒性[22]。

由于AML靶抗原异质性表达，CAR-T细胞疗法靶向单一抗原可能不足以完全清除AML细胞。CD19 CAR-T细胞治疗后疾病复发已经被普遍报道。这种局限性可能需要通过CAR靶向两种或多种肿瘤抗原来解决[8],[23]。靶向CLL-1和其他AML较特异抗原的类似联合可能是一种实用的方法。TIM-3、CD96、CD123等可能可以作为双靶点CAR靶向的第二靶标，从而拓宽易感肿瘤细胞群[24]。

总之，我们成功制备出活性良好的CLL-1 CAR-T细胞，初步研究显示其具有特异性靶向杀伤AML细胞的作用。CLL-1在AML细胞中表达较为特异，其血液系统外毒性较低，可能是AML细胞治疗中较优的靶点之一；此外，该靶点目前在国内外应用较少，进一步的临床试验探索可能会为AML的细胞免疫治疗带来突破。
